# Supplementary material for: Enhancing STED microscopy via fluorescence lifetime unmixing and filtering in two-species SPLIT-STED
Source: Methods Microsc. 2025 Dec 15;3(1):27–41. doi: 10.1515/mim-2025-0026 (PMC13135033; doi:10.1515/mim-2025-0026)
Supplement: Supplementary file 1 — Supplementary Material Details [file j_mim-2025-0026_suppl_001.pdf]

Supplementary Material

Primary Antibodies				
**Antibody**	**Supplier**	**Catalogue no.**	**Dilution**	**Ref.**
Mouse anti-PSD95	Abcam	MA1-045	1 : 500	[1], [2]
Mouse anti-Bassoon	Enzo	ADI-VAM-PS003	1 : 500	[2]–[4]
Rabbit anti-Bassoon	Synaptic Systems	141003	1 : 500	[2], [3]
Rabbit anti-Homer1	Synaptic Systems	160003	1 : 500	[5]
Mouse anti-β2-Spectrin	BD Biosciences	612563	1 : 250	[6], [7]
Mouse anti-α-Tubulin	Sigma-Aldrich	T5168	1 : 500	[8]

Secondary Antibodies			
**Antibody**	**Supplier**	**Catalogue no.**	**Dilution**
Goat anti-Rabbit CF594	Sigma-Aldrich	SAB4600407	1 : 500
Goat anti-Mouse CF594	Sigma-Aldrich	SAB4600321	1 : 500
Goat anti-Rabbit STAR ORANGE	Abberior	STORANGE-1002	1 : 250
Goat anti-Mouse STAR ORANGE	Abberior	STORANGE-1001	1 : 250
Goat anti-Rabbit STAR 635P	Abberior	ST635P-1001	1 : 250
Goat anti-Mouse Alexa Fluor 647	Jackson Immunoresearch	115-605-166	1 : 250

Table S1: Antibodies used for immunostaining

	Red channel	
**Parameter**	**Confocal-FLIM**	**STED-FLIM**
**Excitation wavelength**	561 nm	561 nm
**Excitation power**	2.6 µW	2.6 µW
**Depletion wavelength**		775 nm
**Depletion powers**		44, 88, 132, 176 mW
**Detection wavelengths**	605-625 nm	605-625 nm
**Detection time bins**	250 bins of 80 ps	250 bins of 80 ps
**Pixel dwelltime**	15 µs	15 µs
**Line steps**	6	12 or 25 (Fig. 3)
**Pixel size**	20 nm	20 nm
**Pinhole size**	1.0 A.U.	1.0 A.U.

	Far-red channel	
**Parameter**	**Confocal-FLIM**	**STED-FLIM**
**Excitation wavelength**	640 nm	640 nm
**Excitation power**	1 µW	2 µW
**Depletion wavelength**		775 nm
**Depletion powers**		22,44,66,88 mW
**Detection wavelengths**	650-720 nm	655-720 nm
**Detection time bins**	250 bins of 80 ps	250 bins of 80 ps
**Pixel dwelltime**	15 µs	15 µs
**Line steps**	8	10
**Pixel size**	20 nm	20 nm
**Pinhole size**	1.0 A.U.	1.0 A.U.

	Far-red channel, live-cell	
**Parameter**	**Confocal-FLIM**	**STED-FLIM**
**Excitation wavelength**	640 nm	640 nm
**Excitation power**	2.5 µW	3.6 µW
**Depletion wavelength**		775 nm
**Depletion powers**		22,44,66,88,102,132,176 mW
**Detection wavelengths**	650-720 nm	655-720 nm
**Detection time bins**	250 bins of 80 ps	250 bins of 80 ps
**Pixel dwelltime**	15 µs	15 µs
**Line steps**	3	16
**Pixel size**	25 nm	25 nm
**Pinhole size**	1.0 A.U.	1.0 A.U.

Table S2: Imaging parameters for acquisition of FLIM images

Single-species SPLIT-STED	
**Name**	**Description**
P_n	Confocal-only reference point. Confocal-FLIM phasor's centroid or its closest point on the semi-circle
P_l	Reference point for short lifetime component (Limiting point). (g=1,s=0)
P_1	Point used as limit where f_1=0. Centroid of the first cluster of the STED-FLIM phasor
P_2	Point used as limit where f_1=1. Centroid of the second cluster of the STED-FLIM phasor
**Two-species STED-FLIM**	
**Name**	**Description**
P_1	Reference point for the first fluorophore. Centroid of the STED-FLIM phasor of a single-species image
P_2	Reference point for the second fluorophore. Centroid of the STED-FLIM phasor of a single-species image
**Two-species SPLIT-STED**	
**Name**	**Description**
P_1	Reference point for the first fluorophore. Centroid of the Confocal-FLIM phasor of a single-species image
P_2	Reference point for the second fluorophore. Centroid of the Confocal-FLIM phasor of a single-species image
P_3	Reference point for the short lifetime component. Point on the semi-circle, closest to P_4
P_4	Intersection point of the linear trajectories of both fluorophores, obtained by fitting lines through the centroids of single-species STED-FLIM images acquired at three increasing STED depletion powers

Table S3: Definitions of phasor reference points

Posthoc Dunn's test (p-values) Two-species STED-FLIM vs Two-species SPLIT-STED		
**Depletion Power**	**F1**	**F2**
44 mW	$\mathbf{2.04 \times 10^{-22}}$	$\mathbf{9.14 \times 10^{-8}}$
88 mW	$\mathbf{1.47 \times 10^{-19}}$	$\mathbf{5.21 \times 10^{-14}}$
132 mW	$\mathbf{1.343 \times 10^{-40}}$	$\mathbf{1.04 \times 10^{-16}}$
176 mW	$\mathbf{1.98 \times 10^{-29}}$	$\mathbf{1.22 \times 10^{-28}}$

Table S4: Comparison of NanoJ-SQUIRREL error values for two-species STED-FLIM and two-species SPLIT-STED on the synthetic dataset for different depletion powers. The p-values are obtained by a posthoc Dunn's test following a Kruskal–Wallis H test to compare the distributions of each method at each depletion power (Methods). The main results are reported in Figure 3E. Bold: significant difference between groups ($p < 0.05$)

	Posthoc Dunn's test (p-values)			
	Input vs Two-species STED-FLIM		Input vs Two-species SPLIT-STED	
**Depletion Power**	F_1	F_2	F_1	F_2
44 mW	$\mathbf{1.45 \times 10^{-16}}$	$\mathbf{2.32 \times 10^{-18}}$	$\mathbf{1.58 \times 10^{-28}}$	$\mathbf{3.38 \times 10^{-11}}$
88 mW	$\mathbf{1.56 \times 10^{-37}}$	$\mathbf{1.98 \times 10^{-9}}$	$\mathbf{5.07 \times 10^{-39}}$	$\mathbf{6.42 \times 10^{-26}}$
132 mW	$\mathbf{1.39 \times 10^{-22}}$	3.53×10^{-1}	$\mathbf{3.43 \times 10^{-50}}$	$\mathbf{1.21 \times 10^{-33}}$
176 mW	$\mathbf{3.23 \times 10^{-26}}$	1.26×10^{-1}	$\mathbf{4.29 \times 10^{-38}}$	$\mathbf{5.07 \times 10^{-52}}$

	Posthoc Dunn's test (p-values)	
	Two-species STED-FLIM vs Two-species SPLIT-STED	
**Depletion Power**	F_1	F_2
44 mW	$\mathbf{4.82 \times 10^{-3}}$	$\mathbf{2.63 \times 10^{-53}}$
88 mW	7.92×10^{-1}	$\mathbf{2.33 \times 10^{-61}}$
132 mW	$\mathbf{3.07 \times 10^{-7}}$	$\mathbf{9.70 \times 10^{-39}}$
176 mW	$\mathbf{4.56 \times 10^{-6}}$	$\mathbf{5.77 \times 10^{-30}}$

Table S5: Comparison of spatial resolutions obtained on the synthetic dataset with two-species STED-FLIM and two-species SPLIT-STED for different depletion powers. The p-values are obtained by a posthoc Dunn's test following a Kruskal–Wallis H test to compare the distributions of each method at each depletion power (Methods). The main results are reported in Figures 3F and S14. Bold: significant difference between groups ($p < 0.05$)

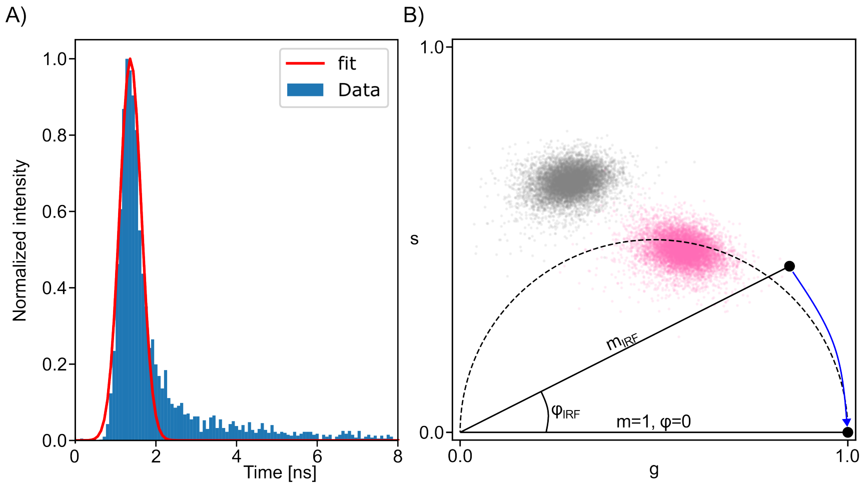

Fig. S1: **Calibration of phasor plots using IRF measurement** (A) Measured histogram from image of gold bead (blue) and gaussian fit (red) used to measure the IRF's FWHM. (B) Phasor distribution of a Confocal-FLIM image of PSD95 STAR ORANGE before (grey) and after (pink) calibration based on a measurement of the IRF.

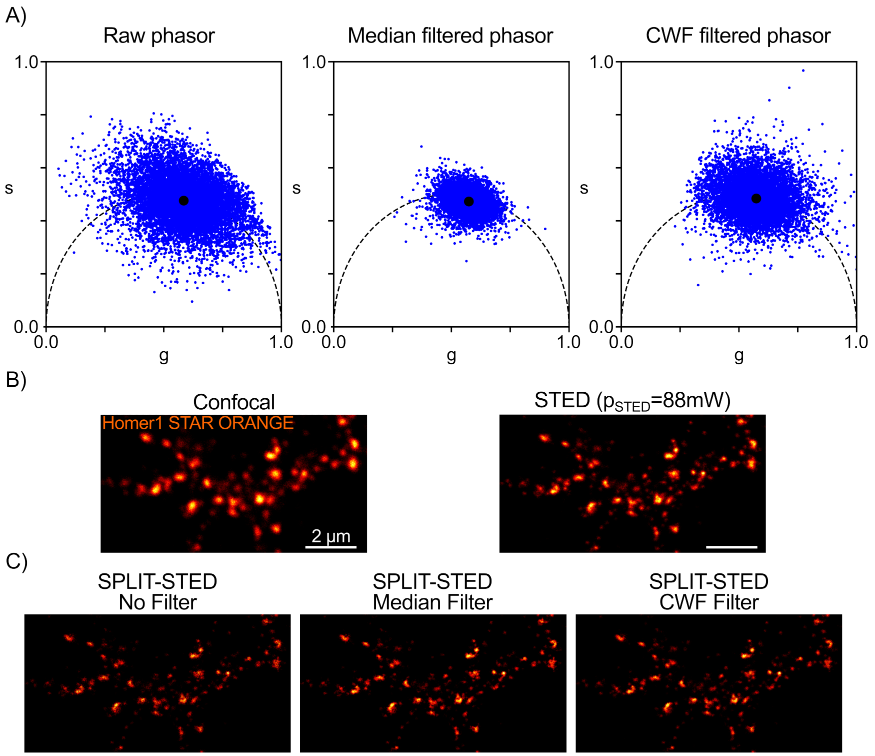

Fig. S2: **Filtering methods in phasor space** (A) Raw phasor distribution of a Confocal-FLIM image of Homer1 STAR ORANGE before filtering (left), with Median filtering (middle) and with CWF filtering (right). (B) Input intensity images of Homer1 STAR ORANGE confocal (left) and STED (right). (C) Comparison of SPLIT-STED images for the three filtering approaches shown in (A).

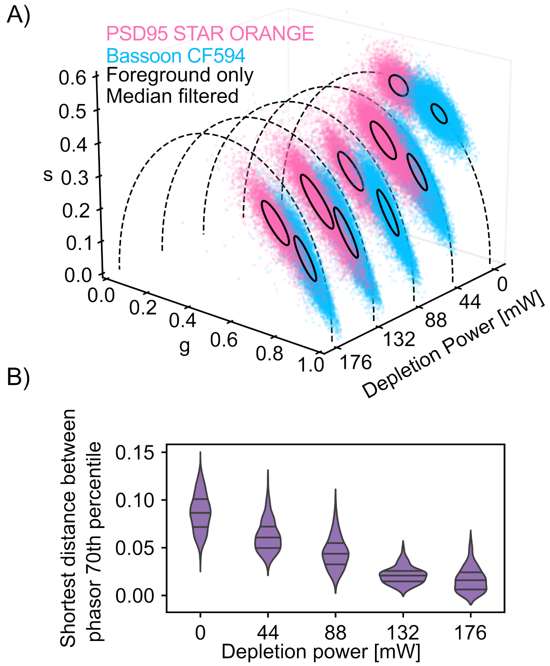

Fig. S3: **Phasor distributions of STED-FLIM images acquired with varying depletion powers**

(A) Representative phasor distributions from single-species images acquired with varying depletion powers. When plotted in the same phasor space, the fluorescence lifetime of Bassoon-CF594 (blue) and PSD95-STAR ORANGE (pink) show increased ellipticity and overlap for increasing STED depletion power. Ellipses (black) represent the 70th percentile of the covariance distribution. (B) Violin plot of the shortest distance between the borders of 70th percentile ellipses. The distance was calculated for each synthetic pair of single-species STED-FLIM images from PSD95-STAR ORANGE and Bassoon CF594. The STED depletion laser significantly affects the shortest distance between the lifetime distributions for all depletion powers when compared to the confocal-FLIM lifetime distributions (p-value from Posthoc Dunn <0.001, ***). Horizontal lines are quartiles.

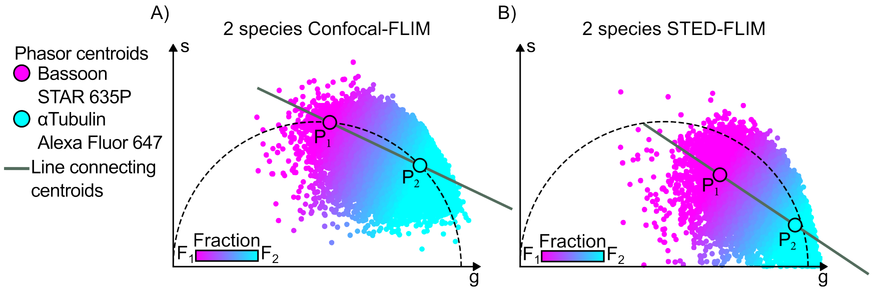

Fig. S4: **Generation of the reference lines for two-species Confocal- and STED-FLIM** The centroids of the lifetime distributions in phasor space from single species images of Bassoon STAR 635P (pink) and αTubulin Alexa Fluor 647 (blue) are used to define the pure species reference points P_1 and P_2 on the universal semicircle. The reference line for (A) two-species Confocal-FLIM and (B) two-species STED-FLIM is built using the P_1 and P_2 reference points. Each point of the phasor distribution of the two-species image is projected onto the reference line. A fraction value is assigned to each point based on its position on the line (color-code).

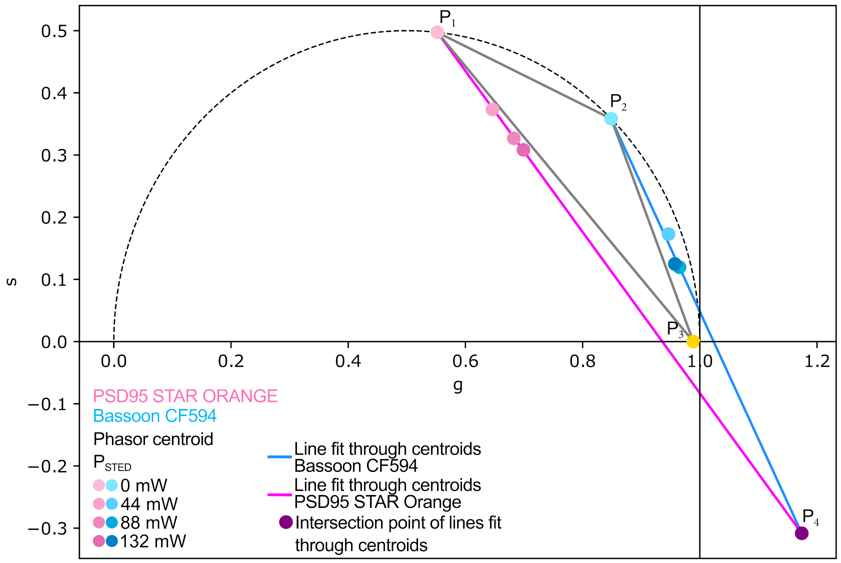

Fig. S5: **Generation of a reference triangle for two-species SPLIT-STED** The centroids of the lifetime distributions in phasor space from single species confocal images of PSD95 STAR ORANGE (pink) and Bassoon CF594 (blue) are used to define the pure species reference points P_1 and P_2 on the universal semicircle. A linear fit on the centroid of the lifetime distributions from STED-FLIM images acquired at different depletion powers is used to define an intersection between both trajectories (P_4). The reference point (P_3) for the third fraction F_3 is defined as the nearest point to P_4 within the universal semicircle. The reference triangle for two-species SPLIT-STED is built using the P_1, P_2 and P_3 reference points.

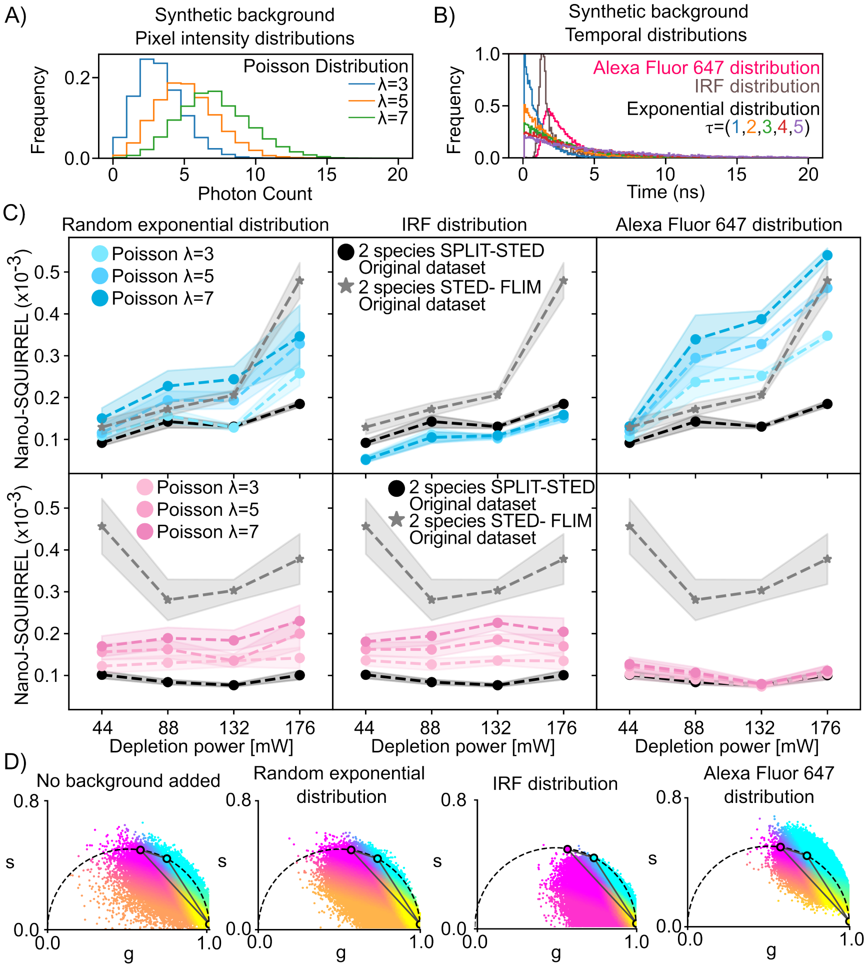

Fig. S6: **Effect of added synthetic background on two-species SPLIT-STED unmixing accuracy**(Continued on next page)

Fig. S6: Synthetic images were generated from pairs of single-species images of Bassoon-CF594 (20 images, 5 per depletion power) and PSD95-STAR ORANGE (20 images, 5 per depletion power). Prior to unmixing, synthetic background was added to the FLIM images: A) background intensity was sampled from one of three Poisson distributions ($\lambda = 3, 5, 7$), and B) temporal bins were selected by sampling from one of three temporal distributions : 1) random exponential distribution with $\tau = 1, 2, 3, 4, 5$ ns, 2) the IRF distribution, and 3) the distribution of Alexa Fluor 647 to simulate crosstalk with another fluorophore. C) The unmixing error, measured with NanoJ-SQUIRREL, increases for both fractions with the addition of random exponential background (left), increases only for fraction 2 (fluorophore with shorter lifetime) with IRF background (middle), and increases only for fraction 1 (fluorophore with longer background) with Alexa Fluor 647 background (right). Circles and dotted lines represent the mean, and shaded areas the SEM, across the images in the dataset. Black and grey curves show the performance without added background using two-species SPLIT-STED and two-species STED-FLIM, on the original dataset respectively. D) Phasor distribution of a representative synthetic image pair (P_{STED}=132 mW), color-coded by the fraction determined with two-species SPLIT-STED for $\lambda = 5$, illustrates how different temporal dynamics of background shift the phasor distribution in distinct directions.

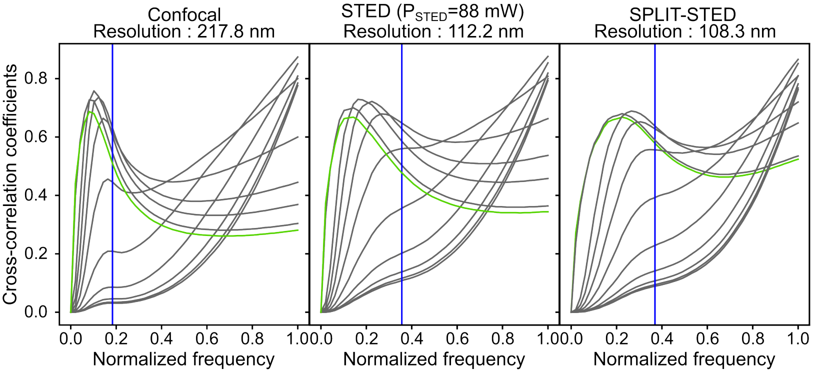

Fig. S7: **Decorrelation curves obtained for resolution estimation** of Confocal-FLIM (left), STED-FLIM (middle) and SPLIT-STED (right) images of Homer1 STAR ORANGE (Same image as in Figure S2). Decorrelation function without any high-pass filtering is shown in green and decorrelation functions with high-pass filtering are shown in grey. The cutoff frequency is shown in blue.

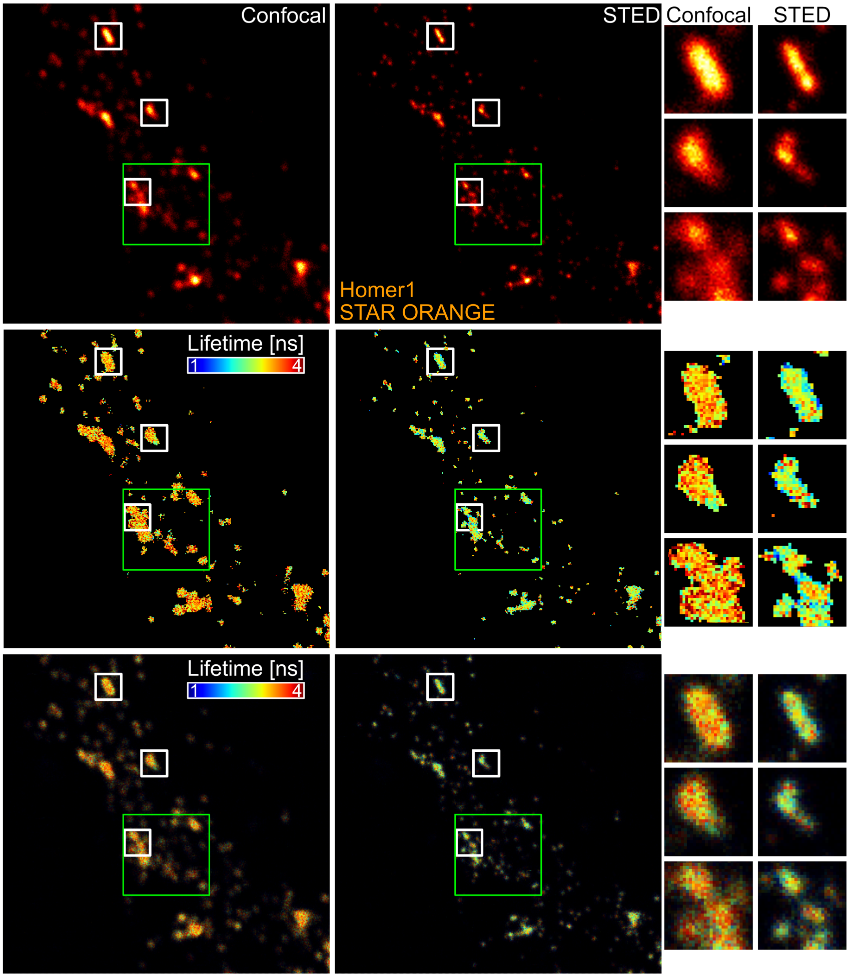

Fig. S8: **Spatial mapping of fluorescence lifetimes in Confocal and STED images** Confocal and STED images of the pre-synaptic protein Homer1 labelled with STAR ORANGE color-coded for Top) Pixel intensity. Middle) mean lifetime obtained with mono-exponential histogram fitting. Bottom) intensity image color-coded using the lifetime distribution. The green box shows the extent of the crop from Figure 1A

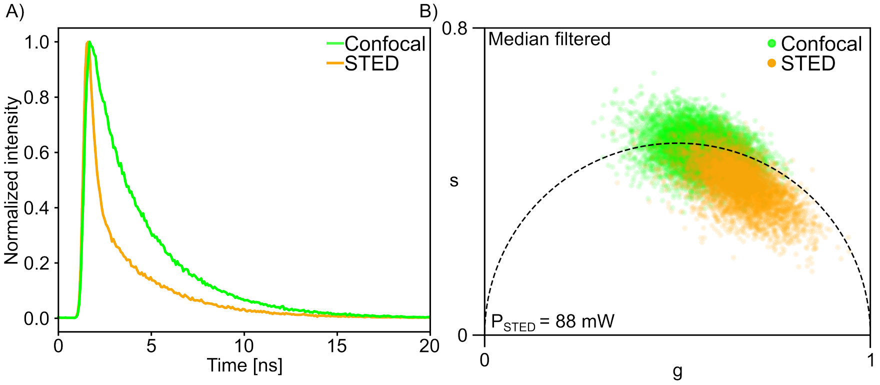

Fig. S9: **Lifetime distribution from a Confocal-(green) and STED-(orange) FLIM image of Homer1 STAR ORANGE.** The distributions are represented using A) the fluorescence decay histograms and (B) the phasor distributions.

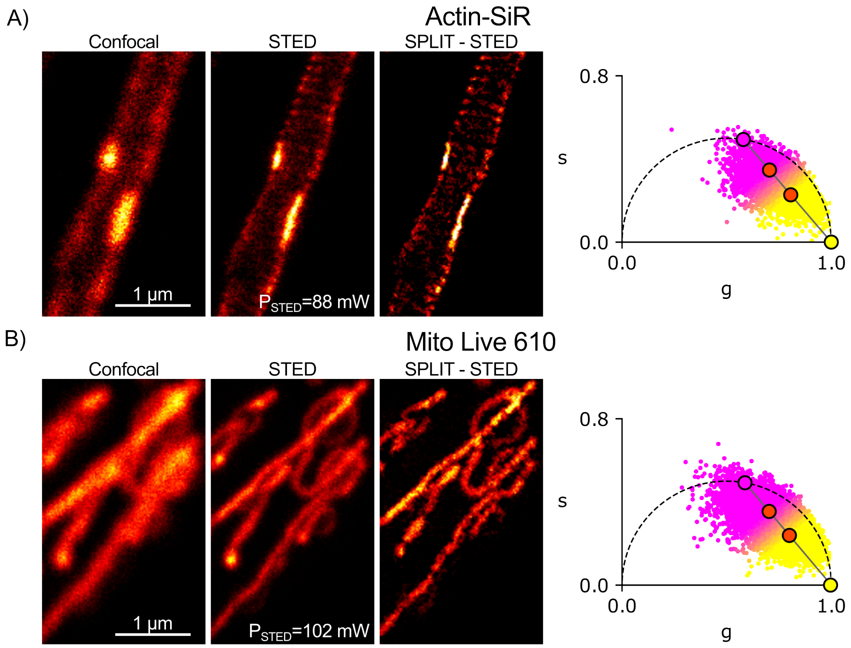

Fig. S10: **Single-species SPLIT-STED in living neurons** Confocal, STED and SPLIT-STED images and associated color-coded phasor distributions for the cytoskeletal protein F-actin (Actin-SiR, P_{STED}=88mW) and B) the mitochondrial membrane (Mito LIVE610, P_{STED}=88mW) in living cultured hippocampal neurons.

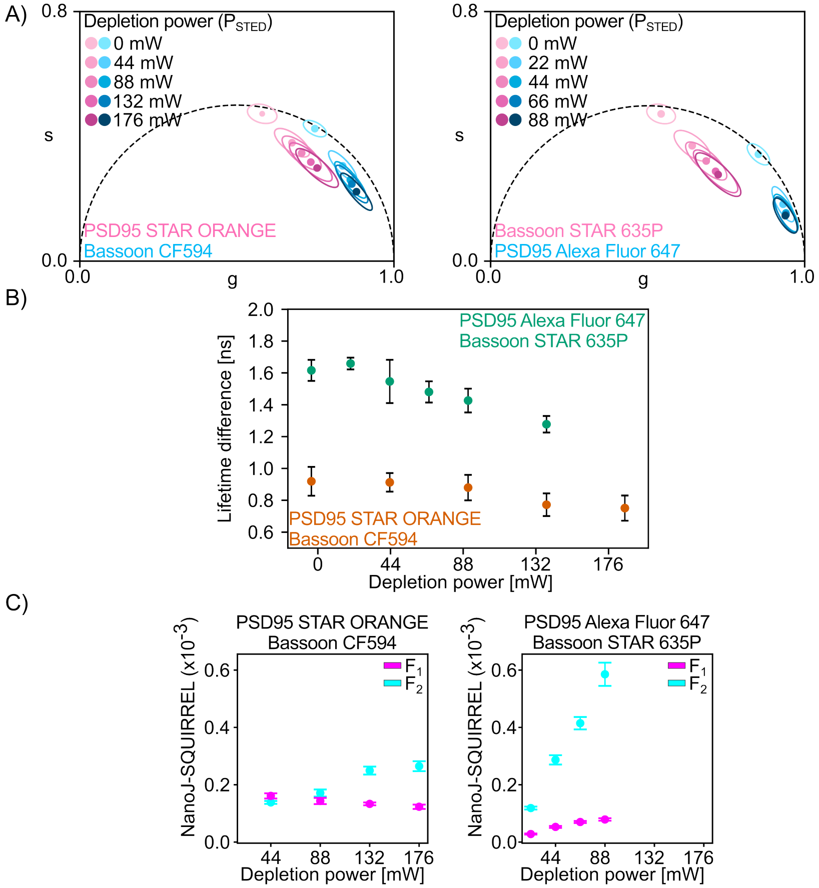

Fig. S11: **Evolution of fluorophore lifetime and unmixing performance with depletion power** (A) Mean centroid and ellipse position of the phasor distributions for Bassoon-CF594 and PSD95-STAR ORANGE (left, same as Figure 2A) and Bassoon-STAR 635P and PSD95-Alexa Fluor 647 (right). (B) Mean lifetime differences decrease with depletion power at different rates for PSD95-STAR ORANGE and Bassoon-CF594 (orange, same data as Figure 2C) compared to Bassoon-STAR 635P and PSD95-Alexa Fluor 647 (green) with the whiskers corresponding to the STD.(C) Unmixing artifacts measured with NanoJ-SQUIRREL increase at a faster rate with depletion power for Bassoon-STAR 635P and PSD95-Alexa Fluor 647 (right) compared to Bassoon-CF594 and PSD95-STAR ORANGE (left) (F_1 - magenta, F_2 - cyan, whiskers are SEM).

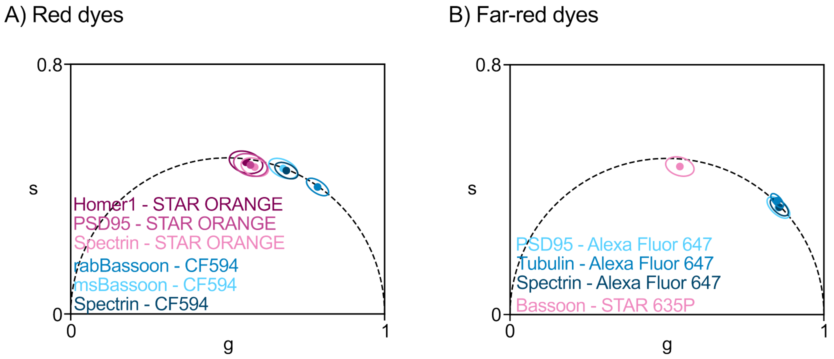

Fig. S12: **Confocal Phasors of different dye-protein pairs**
Mean centroid and ellipse (70th percentile) position of the phasor distributions for Confocal-FLIM images of (A) the red and (B) the far-red imaging channels.

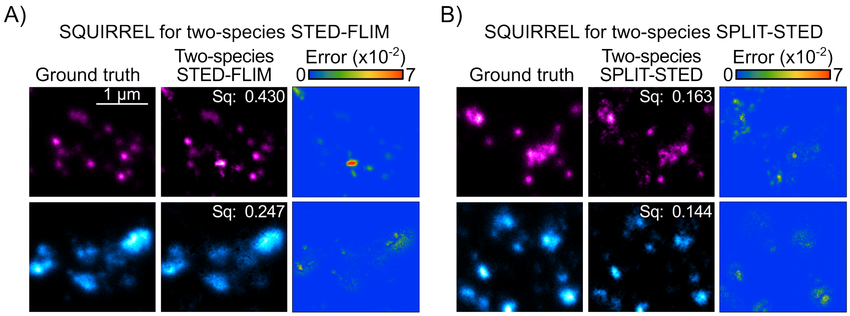

Fig. S13: **Maps of NanoJ-SQUIRREL error score**
Representative maps of NanoJ-SQUIRREL error score for $P_{STED} = 88\,\mathrm{mW}$ obtained on pairs of PSD95 STAR ORANGE (magenta) and Bassoon CF594 (cyan) images from the synthetic dataset for two-species (A) STED-FLIM and (B) SPLIT-STED. Ground truth image (left), unmixed image (middle) and error map (right).

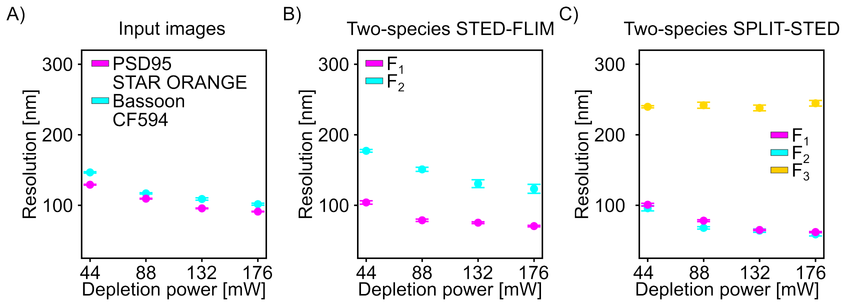

Fig. S14: **Spatial resolution for different depletion powers for two-species STED-FLIM and SPLIT-STED**. The spatial resolution is measured on the synthetic dataset using a decorrelation approach for the synthetic ground truth (A), the unmixed STED-FLIM (B) and SPLIT-STED images (C). SPLIT-STED improves the spatial resolution for the fraction F_1 and F_2 and filters out the low resolution F_3 component. Synthetic images were created for pairs of Bassoon-CF594 (56 images, 15- 44 mW; 14- 88 mW;14- 132 mW and 13 - 176 mW) and PSD95-STAR ORANGE (40 images, 10 images per depletion power). Shown is the mean and SEM for each depletion power. Panel (C) is the same as Figure 3F.

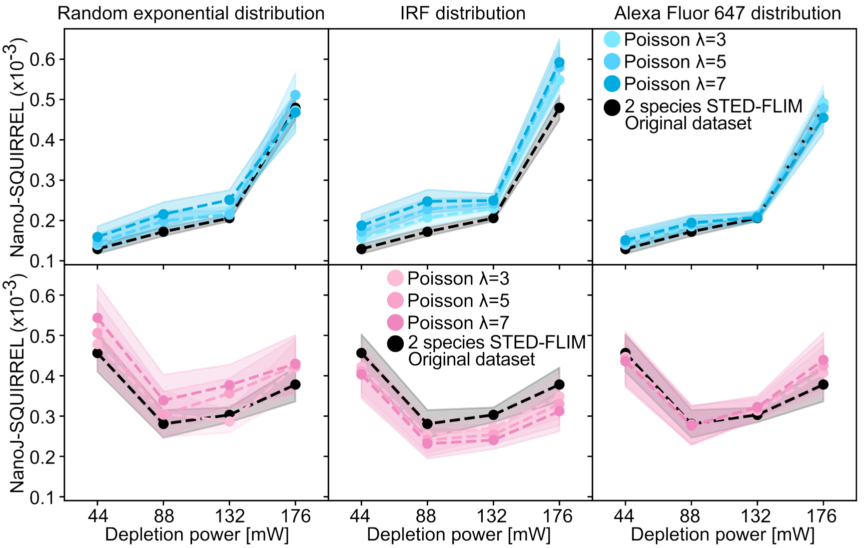

Fig. S15: **Effect of added synthetic background on two-species STED-FLIM unmixing accuracy** The unmixing error of two-species STED-FLIM of the same synthetic image dataset with added synthetic background as Figure S6 measured with NanoJ-SQUIRREL. Circles and dotted lines represent the mean, and shaded areas the SEM, across the images in the dataset. Black curves show the performance without added background using two-species STED-FLIM, on the original dataset.

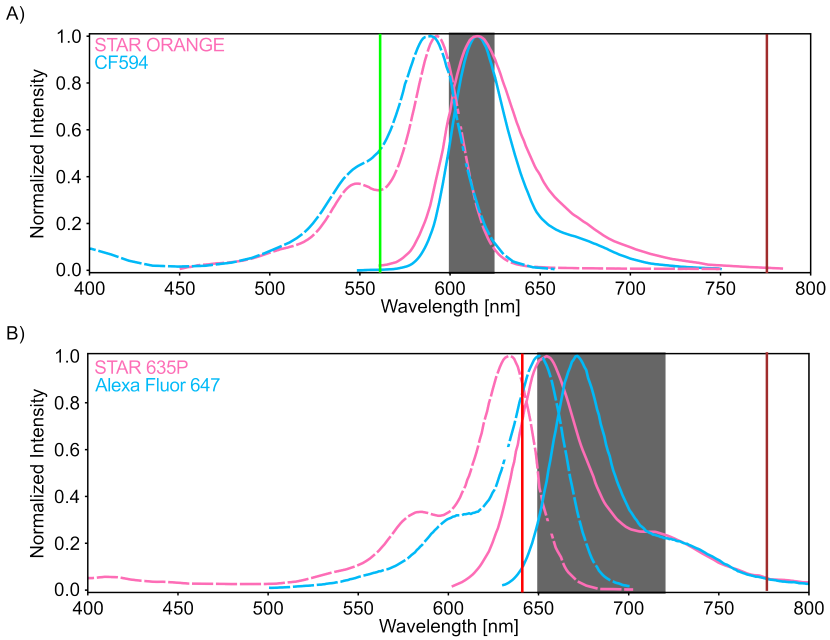

Fig. S16: **Fluorescence spectra for selected dye pairs**
Excitation (dashed line) and emission (full line) spectra of A) The red dye pair CF594 (blue) STAR ORANGE (pink) and B) the far-red dye pair Alexa Fluor 647 (blue) STAR 635P (pink). Laser lines are shown as solid vertical lines and detection bins as shaded grey rectangles.

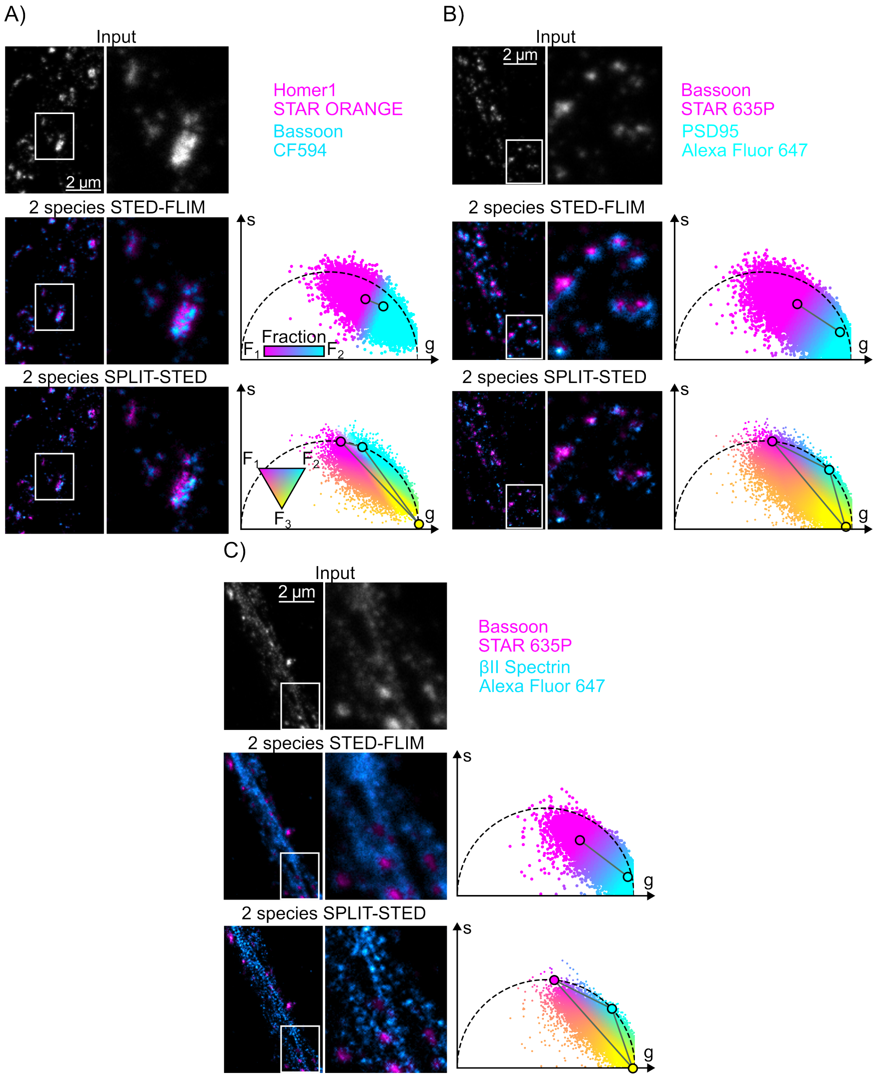

Fig. S17: **Unmixing of different neuronal protein pairs in real images** Two-species SPLIT-STED is applied to different pairs of neuronal proteins labelled with red and far-red emitting fluorophores. (A) Bassoon CF594 and Homer1 STAR ORANGE imaged with $P_{STED} = 132\,\mathrm{mW}$ (B) PSD95 Alexa Fluor 647 and Bassoon STAR 635P imaged with $\mathrm{P_{STED}} = 44\mathrm{mW}$ and (C) βII-Spectrin Alexa Fluor 647 and Bassoon STAR 635P imaged with $\mathrm{P_{STED}} = 44\mathrm{mW}$. Intensity image (top) and unmixed images with two-species STED-FLIM (middle) and two-species SPLIT-STED approaches (bottom). Phasor plots color-coded by the assigned fraction at each pixel are shown for both methods.

References

[1] L. Ladépêche *et al.*, "NMDA Receptor Autoantibodies in Autoimmune Encephalitis Cause a Subunit-Specific Nanoscale Redistribution of NMDA Receptors," *Cell Reports*, vol. 23, no. 13, pp. 3759–3768, Jun. 2018. DOI: 10.1016/j.celrep.2018.05.096.

[2] T. Wiesner *et al.*, "Activity-Dependent Remodeling of Synaptic Protein Organization Revealed by High Throughput Analysis of STED Nanoscopy Images," *Frontiers in Neural Circuits*, vol. 14, Oct. 2020. DOI: 10.3389/fncir.2020.00057.

[3] A. Dani, B. Huang, J. Bergan, C. Dulac, and X. Zhuang, "Superresolution Imaging of Chemical Synapses in the Brain," *Neuron*, vol. 68, no. 5, pp. 843–856, Dec. 2010. DOI: 10.1016/j.neuron.2010.11.021.

[4] A.-H. Tang, H. Chen, T. P. Li, S. R. Metzbower, H. D. MacGillavry, and T. A. Blanpied, "A trans-synaptic nanocolumn aligns neurotransmitter release to receptors," *Nature*, vol. 536, no. 7615, pp. 210–214, Aug. 2016. DOI: 10.1038/nature19058.

[5] T. Lagache *et al.*, "Mapping molecular assemblies with fluorescence microscopy and object-based spatial statistics," *Nature Communications*, vol. 9, no. 1, p. 698, Feb. 2018. DOI: 10.1038/s41467-018-03053-x.

[6] K. Xu, G. Zhong, and X. Zhuang, "Actin, Spectrin, and Associated Proteins Form a Periodic Cytoskeletal Structure in Axons," *Science*, vol. 339, no. 6118, pp. 452–456, Jan. 2013. DOI: 10.1126/science.1232251.

[7] F. Lavoie-Cardinal *et al.*, "Neuronal activity remodels the F-actin based submembrane lattice in dendrites but not axons of hippocampal neurons," *Scientific Reports*, vol. 10, no. 1, p. 11 960, Jul. 2020. DOI: 10.1038/s41598-020-68180-2.

[8] A. Durand *et al.*, "A machine learning approach for online automated optimization of super-resolution optical microscopy," *Nature Communications*, vol. 9, no. 1, p. 5247, Dec. 2018. DOI: 10.1038/s41467-018-07668-y.

List of Acronyms

FWHM Full Width at Half Maximum

CWF Complex Wavelet Filter

SEM Standard Error of the Mean

STD Standard Deviation
